# Identification and Characterization of Regulatory Pathways Controlling Dormancy Under Lower Temperature in Alfalfa (*Medicago sativa* L.)

**DOI:** 10.3389/fpls.2022.872839

**Published:** 2022-06-02

**Authors:** Jingfu Liu, Tiemei Wang, Yinyin Weng, Bei Liu, Qiu Gao, Wei Ji, Zhuanling Wang, Yingwei Wang, Xiqing Ma

**Affiliations:** ^1^College of Grassland Science and Technology, China Agricultural University, Beijing, China; ^2^College of Grassland Science, Beijing Forestry University, Beijing, China; ^3^National Animal Husbandry Service, Beijing, China; ^4^Beijing Botanical Garden, Institute of Botany, Chinese Academy of Sciences, Beijing, China

**Keywords:** alfalfa, dormancy, low temperature, transcriptome, flavonoids

## Abstract

Alfalfa (*Medicago sativa* L.), a kind of high-quality perennial legume forage, is widely distributed in the northern regions of China. In recent years, low temperatures have frequently occurred and limited alfalfa productivity and survival in early spring and late fall. However, the underlying molecular mechanisms of alfalfa response to cold tolerance are not well-documented. In this study, dormancy and non-dormancy alfalfa standard varieties were characterized under low-temperature stress. Our analysis revealed that plant height of the dormancy genotype was strongly inhibited by low temperature; flavonoids content, and higher expression of flavonoids biosynthesis genes (*chalcone synthase, leucoanthocyanidin dioxygenase*, and *flavonoid 3'-monooxygenase*) may play essential roles in response to low-temperature stress in dormancy genotype alfalfa. Further analyses revealed that receptor-like kinase family genes (such as *cysteine-rich RLK10, lectin protein kinase*, and *S-locus glycoprotein like kinase*), RNA and protein synthesis genes (*RNA polymerases, ribosomal protein*, and *protein phosphatase 2C family protein*), and proteasome degradation pathway genes (such as *F-box family protein, RING/U-box superfamily protein*, and *zinc finger family protein*) also highly upregulated and contributed to cold tolerance phenotype in dormancy genotype alfalfa. This will provide new insights into future studies for cold tolerance in alfalfa and offer new target genes for further functional characterization and genetic improvement of alfalfa.

## Introduction

Alfalfa (*Medicago sativa* L.), as a kind of high-quality perennial legume forage, occupies an important position in animal husbandry with great agronomic and environmental traits, and is widely distributed in northern parts of China (Kumar, 2011; Singer et al., 2018). In recent years, low temperature (LT) has occurred frequently and limits alfalfa productivity and survival in early spring and late fall, especially for higher dormancy alfalfa cultivars (Brouwer et al., 2000; Kanchupati et al., 2017). However, the underlying molecular mechanisms of alfalfa response to cold tolerance are not well-documented.

Flavonoids are a major class of secondary plant metabolites that affect plant growth and development. The biosynthesis of flavonoids in plants involves chalcone synthase (*CHS*), chalcone isomerase (*CHI*), flavanone 3-hydroxylase (*F3H*), flavonol synthase (*FLS*), and anthocyanidin synthase (*ANS*) (Schulz et al., 2016). Past cold tolerance studies demonstrated that low temperature strongly increased flavonoid content, resulting from the enhanced expression of flavonoid biosynthesis genes (Cheng et al., 2014). For instance, *CHS, ANS*, and *UDP-glucosyl transferase family proteins* were all induced by cold stress in both blood orange (*Citrus sinensis*) and strawberry (*Fragaria* × *ananassa*; Crifò et al., 2011; Koehler et al., 2012). Meanwhile, receptor-like kinase family proteins (RLKs) also play critical roles in plant response to a variety of internal and external stimuli including cold stress (Lim et al., 2015; Wu et al., 2016). For example, TaCRK68-A, a cysteine-rich receptor-like kinase, had been shown to enhance plant tolerance against cold stress in bread wheat (*Triticum aestivum*; Shumayla et al., 2019). Overexpression of GsLRPK, a leucine-rich repeat receptor-like protein kinase (LRR-RLK), in Arabidopsis strengthens its cold tolerance (Yang et al., 2014). Proteins regulating RNA and protein synthesis are very important for adjusting and determining the final levels of mRNAs and proteins, and are actively involved in plant adaption or response to environmental stresses (Nakaminami and Seki, 2018). Rbm3, a glycine-rich RNA-binding protein that is induced by cold temperature, has been evidenced to enhance global protein synthesis *via* binding 60s ribosomal subunits in N2a cells (Dresios et al., 2005). Furthermore, the ubiquitin–proteasome system plays an essential role in enabling plants to alter their proteome in order to effectively and efficiently perceive and respond to environmental stresses including low temperature. The ubiquitin–proteasome system involves the sequential actions of three enzymes: E1 (ubiquitin-activating enzyme), E2 (ubiquitin-conjugating enzyme), and E3 (ubiquitin-protein ligase enzyme), and is followed by substrate degradation *via* 26S proteasome (Stone, 2014). The overexpression of HOS1 (high expression of osmotically responsive gene (1), a RING finger ubiquitin E3 ligase, substantially reduced ICE1 (inducer of CBF expression (1) protein level and increased Arabidopsis sensitivity to freezing stress (Chinnusamy et al., 2007). However, whether their homologs participate in cold tolerance regulation and dormancy in alfalfa is not clear.

Dormancy, an adaptive characteristic response caused by shortened day-length and decreased temperature in autumn, slows alfalfa growth, or induces alfalfa creeping stems, which may cause more resources to be reallocated from growth to cold tolerance (Brummer et al., 2000). Further investigation indicated that more soluble sugars, proline, glycine, and antioxidants accumulation in dormancy alfalfa were enhanced to alleviate the damage caused by cold tolerance (Liu et al., 2019). Based on our previous study, low temperature (10°C) is critical for differentiation between dormancy and non-dormancy standard varieties depending on the regrowth of alfalfa (Xu and Lu, 2010; Zhang et al., 2015). However, little is known about the underlying molecular mechanism in alfalfa dormancy response to low temperature. In this study, multiple methods such as phenotypical, physiological, and transcriptome analyses were performed to reveal the underlying cold signaling network involved in dormancy and non-dormancy alfalfa varieties. Based on our study, we hypothesize that flavonoids biosynthesis, kinase signaling pathway, RNA and protein synthesis, and ubiquitin-dependent protein degradation pathway all contribute to dormancy under low-temperature stress in alfalfa.

## Materials and Methods

### Plant Materials and Growth Conditions

Alfalfa standard dormancy variety Maverick (FD score = 1, D) and non-dormancy variety UC-1456 (FD score = 11, ND) were used in this research based on our previous studies in China (Xu and Lu, 2010). Each genotype clonal lines were established in a climate chamber at 25°/23°C (day/night), with a 16-h light/8-h dark photoperiod and 120 μmol m^−2^ s^−1^ light intensity (Ma et al., 2021). Plants were irrigated weekly with half-strength Hoagland nutrient solution (Hoagland and Arnon, 1950) and trimmed weekly for uniformity.

### Treatments and Experimental Design

After 6 months of establishment in the chamber, these clonal lines were used for low-temperature treatments (10°C), three replicates were arranged in a randomized complete block design, each replicate contained three plants with trimmed 20 cm height (from the same clones of the unanimous genotype) grown in individual pots. Under normal growth conditions (as control treatments), these trimmed ND alfalfa genotypes began flowering and samples were taken at 24 days. Plant heights were measured every 3 days, and internode length was measured based on the same position of the upper part of each plant at sampling time.

### Total RNA Extraction, RNA-Seq Library Construction, and Sequencing

Mature leaves from the upper part of 24-d-old alfalfa varieties grown under normal temperature (25°C) or low temperature (10°C) were collected and ground into fine powder in liquid nitrogen. Total RNA was extracted using the Trizol reagent (Invitrogen, USA) according to the manufacturer's protocol. After DNase treatment, RNA samples were quantified using the Agilent Bioanalyzer 2100 system (Agilent Technologies, USA), and 2 μg of total RNA with integrity number (RIN) of ≥8 was used for rRNA depletion. RNA-Seq library was constructed using the NEBNext^®^Ultra™ RNA Library Prep Kit for Illumina^®^ (NEB, USA) following the manufacturer's instructions. The 150-bp paired-end sequencing for all the libraries was performed on an Illumina Hiseq 2000 platform (Illumina, USA). Raw reads were filtered and trimmed using SOAPnuke software v2.1.6 (https://github.com/BGI-flexlab/SOAPnuke), and clean reads were obtained for contigs, transcripts, and unigenes assembly with Trinity software v2.9.0 (https://github.com/trinityrnaseq/trinityrnaseq/releases).

### Functional Annotation of Unigenes and Analysis of Differentially Expressed Genes

Functional annotation of unigenes was aligned to protein database such as non-redundant (NR), Swiss-prot, GO, Clusters of Orthologous Groups (COG), KEGG, eukaryotic Orthologous Groups (KOG), and Protein family (Pfam) with Blastx algorithm (https://www.ncbi.nlm.nih.gov/, *E*-value ≤ 10^−5^). The DESeq R package (v1.10.1; Anders and Huber, 2012) was performed to identify the differentially expressed genes (DEGs) with a twofold change and an adjusted *p* < 0.05. The phytozome *Medicago truncatula* genome database (https://phytozome.jgi.doe.gov/pz/portal.html#!info?alias=Org_Mtruncatula, Mt4.0v1, *E*-value <10^−5^) was used to align the homologs genes for the classification and functional analysis, and the MapMan software (https://mapman.gabipd.org/download, V3.5.1R2) was used for dormancy regulatory network analysis. The Venn graph and heat maps were drawn using TBtools (https://github.com/CJ-Chen/TBtools/releases).

### Determination of Total Flavonoids in Alfalfa

The samples (the same leaves as those used for the RNA-Seq analysis) were dried in an oven at 65°C for the determination of flavonoids content by aluminum chloride method according to Shah and Hossain (2014) with minor modifications. Briefly, 0.02 g of dried and ground plant materials was extracted with 2 ml of 60% ethanol in a centrifugation tube, and then the tubes were shaken for 2 h at 60°C and centrifuged at 10,000 *g* for 10 min at RT. 0.5 ml of supernatant, 0.1 ml of 10% aluminum chloride, 0.1 ml of potassium acetate, and 4.3 ml of double-distilled water were mixed and incubated for 30 min at RT, the absorbance was measured at 415 nm using a spectrophotometer (Hitachi UH5300, Tokyo, Japan). The content of total flavonoids was calculated based on standard curves (y = 5.02x + 0.0007).

### Validation of RNA-Seq by RT-qPCR

Purified RNA samples (the same leaves as those used for the RNA-Seq analysis) were reverse-transcribed using the MLV-Reverse transcriptase (Takara Bio, Inc., Otsu, Japan). Thirteen genes from the DEGs list were used for RT-qPCR assay on a Bio-Rad CFX96 real-time PCR detection system. The detailed information of primer pairs was listed in Supplementary Table 1. The 2^−Δ*ΔCT*^ method (Grabherr et al., 2011) was used to calculate the relative expression level of each gene.

### Statistical Analysis

All data were subjected to the analysis of variance (ANOVA) based on the general linear model of SPSS22.0 software (SPSS Inc., Chicago, IL, USA), and Fisher's least significant difference test (LSD, *p* < 0.05) was used to determine significant differences among treatments according to Ma et al. (2021).

## Results

### Phenotypic Characterization of Dormancy in Alfalfa Under Lower Temperature

**Figure 1:**
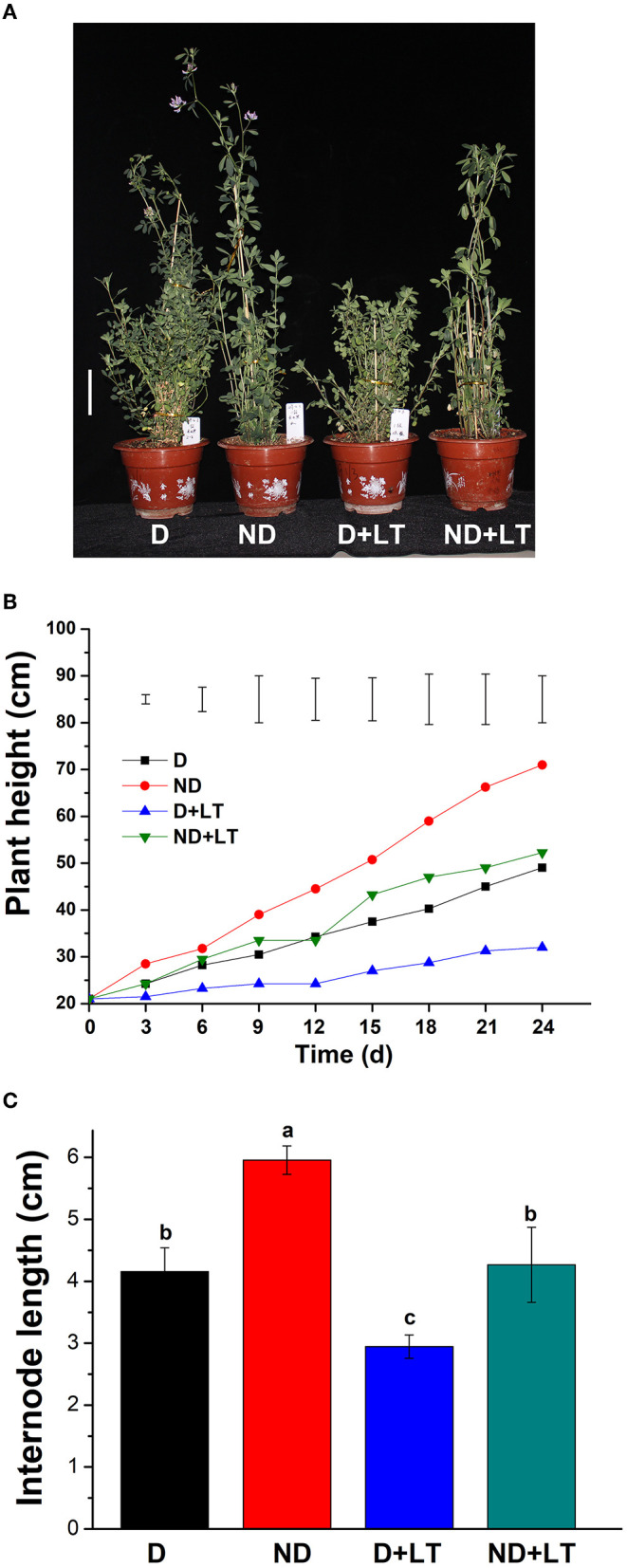
Phenotypic characterization of dormancy and non-dormancy alfalfa cultivars under lower temperature (LT). **(A)** The phenotype of dormancy (D, Maverick) and non-dormancy (ND, UC-1465) alfalfa varieties under normal growth or LT conditions in a growth chamber at sampling time. Scale bar = 10 cm. Plant height **(B)** and internode length **(C)** of D and ND genotypes under normal growth or LT conditions. Vertical bars in **(B)** are least significant difference (LSD) values (*p* ≤ 0.05) (*n* = 9) indicating significant differences among treatments. Columns marked with different letters in **(C)** indicate significant differences among treatments based on the LSD value (*p* ≤ 0.05; *n* = 9).

To understand how low temperature affects alfalfa growth and development, D and ND alfalfa genotypes were trimmed to the same height and continued to grow in a controlled growth chamber. After 24 days of growth under normal conditions, the ND alfalfa genotype began flowering, and the plant height of the ND genotype was 71 ± 0.71 cm. However, the D genotype was still growing with a mean plant height of 49 ± 0.35 cm. Under low temperature, both the D and ND phenotypes are still in the vegetative growth stage. The plant height of the ND genotype was reduced by 37.25% (mean plant height is 52.25 ± 0.88 cm) and the D genotype reduced by 58.65% (mean plant height is 32 ± 1.41 cm) compared with that grown under normal conditions, respectively (Figures 1A,B). Furthermore, under low-temperature treatment, the internode length of ND genotype reduced by 28.40% (4.26 ± 0.20 vs. 5.95 ± 0.07 cm) and that of D genotype reduced by 29.16% (2.94 ± 0.06 vs. 4.15 ± 1.13 cm; Figure 1C). These results indicate that the D alfalfa genotype is more sensitive to low temperature in the aspect of plant height growth compared to the ND phenotype.

### Transcriptome Profiling of D and ND Genotype Alfalfa Under Low Temperature

**Figure 2:**
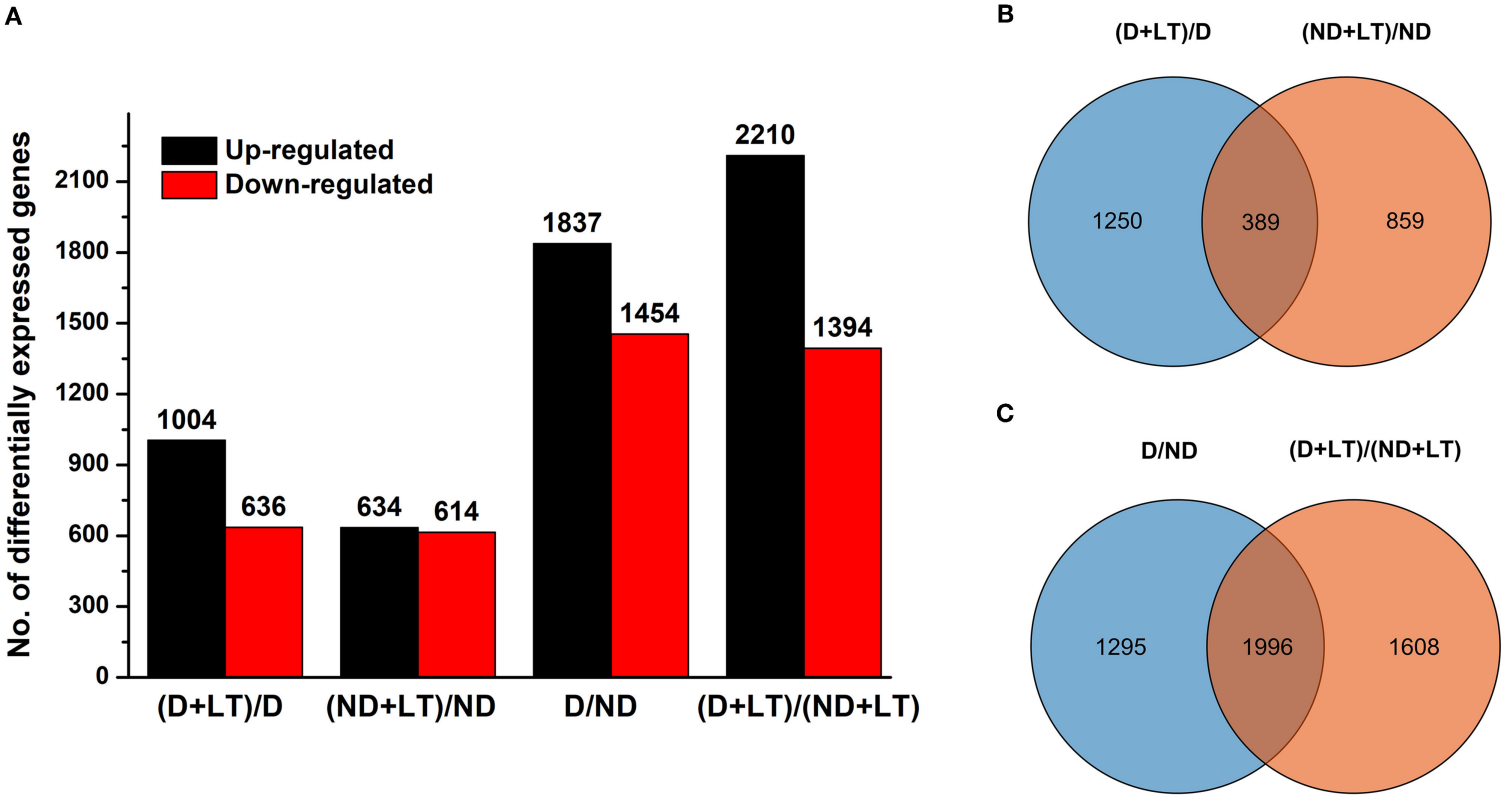
The bar chart **(A)** and Venn diagram **(B,C)** of differentially expressed genes (DEGs) in dormancy (D) and non-dormancy (ND) alfalfa genotype under LT conditions.

To further investigate the molecular basis of different D genotype alfalfa responses to low temperature, we analyzed the transcriptome profile of these genotypes. A total of 95.18 Gb of clean reads were obtained, the Q30 percentage and GC percentages were 93.45 and 42.92%, respectively. A total of 68,834 unigenes were obtained from the assembly (N50 of 1610 bp, mean length of 852.17 bp) and were annotated using seven functional databases such as NR, Swiss-prot, GO, COG, KEGG, KOG, and Pfam with Blastx algorithm (https://www.ncbi.nlm.nih.gov/, *E*-value ≤ 10^−5^). Then, a total of 32,448 coding sequence transcripts (N50 of 2,004 bp, mean length of 1,338 bp) were used for further functional analysis (Table 1). Furthermore, a total of 1,640 (1,004 upregulated/636 downregulated) and 1,248 (634 upregulated/614 downregulated) DEGs were identified in D genotype and ND genotype compared with the control under low temperature, respectively, among which 1,250 and 858 DEGs were exclusively expressed in genotype D and ND, respectively (Figures 2A,B and Supplementary Table 2). In addition, 3,291 (1,837 upregulated/1,454 downregulated) DEGs were identified between D and ND genotype under normal or low-temperature conditions, respectively, among them, 1,295 and 1,608 DEGs were exclusively expressed between D and ND genotype under normal or low-temperature conditions, respectively (Figures 2A,C and Supplementary Table 2). These results indicated that more DEGs were induced in D genotype of alfalfa compared to the ND genotype under low temperature.

**Table 1:** Summary of the transcriptome analysis of leaves in dormancy and non-dormancy alfalfa cultivars under lower temperatures.

**Total clean reads (Gb)**	**95.18**
Q30 bases (%)	93.45
GC content (%)	42.92
Total number of unigenes	68,834
N50 of unigenes (bp)	1,610
Mean length of unigenes (bp)	852.17
Number of transcripts (coding sequence, CDS)	32,448
N50 of transcripts (bp)	2,004
Mean length of transcripts (bp)	1,338

### Response of Flavonoids Biosynthesis to Dormancy in Alfalfa

**Figure 3:**
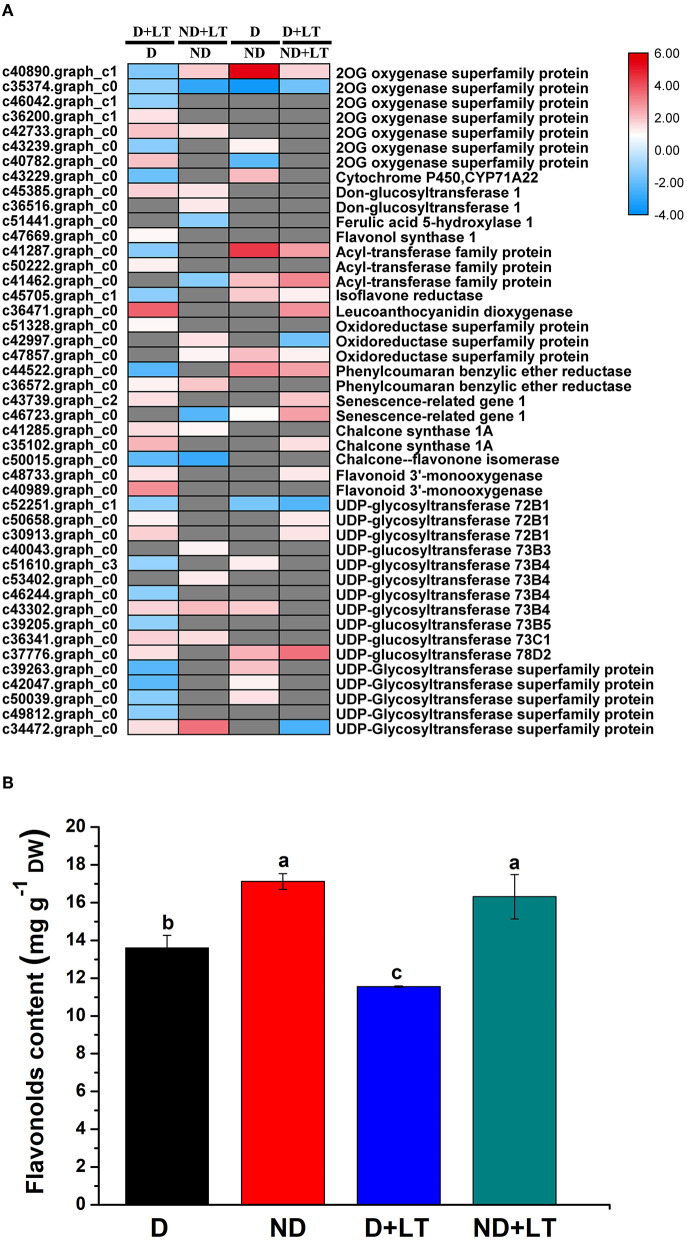
Transcript profiles of flavonoids biosynthesis genes **(A)** and flavonoids content determination **(B)** in different dormancy varieties of alfalfa under LT. **(A)** The color scale indicates log_2_-transformed fold changes in expression levels between dormancy (D) and non-dormancy (ND) genotype under normal growth condition or LT condition. Red, blue, and gray denote upregulation, downregulation, and no change in expression, respectively. **(B)** Flavonoids contents of dormancy (D) and non-dormancy (ND) alfalfa varieties under LT condition. Columns marked with different letters indicate significant differences among treatments based on the LSD value (*p* ≤ 0.05; *n* = 3).

A total of 37 DEGs (20 upregulated/17 downregulated genes) in the D genotype and 18 DEGs (13 upregulated/5 downregulated genes) in the ND genotype involved in flavonoids biosynthesis were significantly enriched compared with the control under low-temperature conditions. At the same time, 18 DEGs (15 upregulated/3 downregulated genes) and 18 DEGs (14 upregulated/4 downregulated genes) involved in flavonoids biosynthesis were significantly enriched in D genotype compared with that of in ND genotype under normal or low-temperature conditions, respectively (Figure 3A and Supplementary Figure 1, Supplementary Table 3). These mainly included genes involved in *2-oxoglutarate and Fe(II)-dependent oxygenase superfamily proteins* (*2OG oxygenases*), *FLS1, leucoanthocyanidin dioxygenase, CHS1A, flavonoid 3'-monooxygenase*, and *UDP-glycosyltransferase*. Furthermore, total flavonoids content was measured. Under low-temperature treatment, flavonoids content in genotype D significantly decreased by 15.07% compared with the control, there was no difference between low temperature and normal growth condition in ND genotype alfalfa (Figure 3B). Altogether, these findings indicated that the D genotype was more sensitive than the ND genotype in response to low-temperature stress, higher upregulation of flavonoids biosynthesis genes, and lower flavonoids content were identified in D genotype alfalfa by low temperature.

### Responses of Receptor-Like Kinase Family Genes to Dormancy

**Figure 4:**
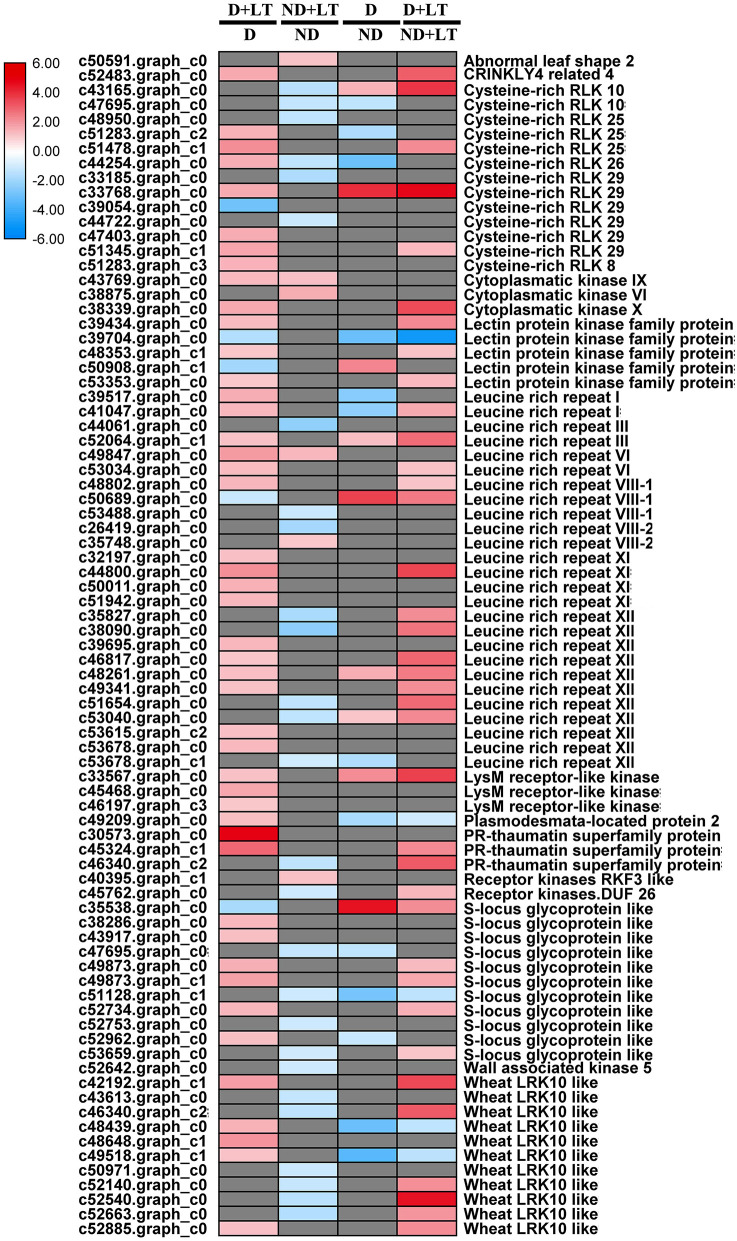
Transcript profiles of receptor kinase signaling in different dormancy varieties of alfalfa under LT. The color scale indicates log_2_-transformed fold changes in expression levels between dormancy (D) and non-dormancy (ND) genotype under normal growth conditions or LT conditions. Red, blue, and gray denote upregulation, downregulation, and no change in expression, respectively.

A total of 51 DEGs (46 upregulated/5 downregulated) in the D genotype and 33 DEGs (6 upregulated/27 downregulated) in the ND genotype involved in the receptor kinase signaling pathway were significantly induced by low temperature compared with the control. Meanwhile, 22 DEGs (9 upregulated/13 downregulated) and 42 DEGs (37 upregulated/5 downregulated) involved in the receptor kinase signaling pathway were significantly enriched in D genotype than in ND genotype under normal or low-temperature conditions, respectively (Figure 4 and Supplementary Figure 2, Supplementary Table 3). These mainly included genes involved in *cysteine-rich RLK10, lectin protein kinase family protein, thaumatin superfamily protein, S-locus glycoprotein-like*, and *wheat LRK10-like kinases*. Taken together, these results revealed that more RLK genes were upregulated in response to low-temperature stress in D genotype than ND genotype alfalfa.

### Responses of RNA and Protein Synthesis Genes to Dormancy

**Figure 5:**
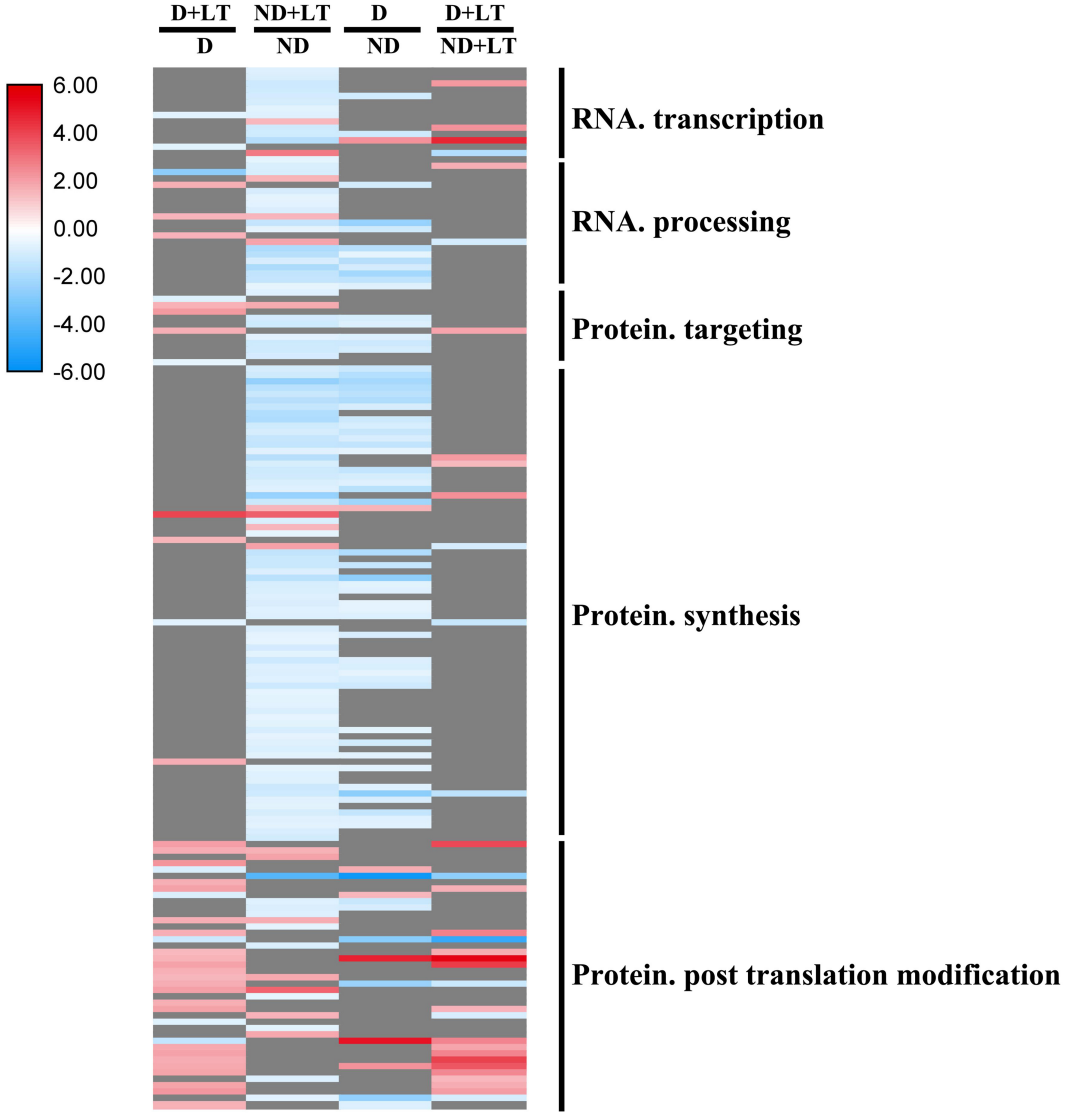
Transcript profiles of RNA and protein synthesis genes in different dominancy varieties of alfalfa under LT. The color scale indicates log_2_-transformed fold changes in expression levels between dormancy (D) and non-dormancy (ND) genotype under normal growth conditions or LT conditions. Red, blue, and gray denote upregulation, downregulation, and no change in expression, respectively.

A total of 44 DEGs (33 upregulated/11 downregulated) in D genotype and 129 DEGs (17 upregulated/112 downregulated) in ND genotype involved in RNA and protein synthesis were significantly enriched in alfalfa grown under low temperature compared with that grown under normal temperature. At the same time, 73 DEGs (9 upregulated/66 downregulated) and 34 DEGs (24 upregulated/10 downregulated) involved in RNA and protein synthesis genes were significantly enriched in D genotype compared with that in ND genotype under normal or low-temperature conditions, respectively (Figure 5 and Supplementary Figure 3, Supplementary Table 3). These mainly include genes involved in *DNA-dependent RNA polymerases, polynucleotide adenylyltransferase family protein, WD-40 repeat protein, ribosomal protein, protein phosphatase 2C family protein*, and *RLKs*. These results further revealed that more RNA and protein synthesis genes were also upregulated in response to low temperature in D genotype than in ND genotype alfalfa.

### Response of Proteasome-Related Genes to Dormancy

**Figure 6:**
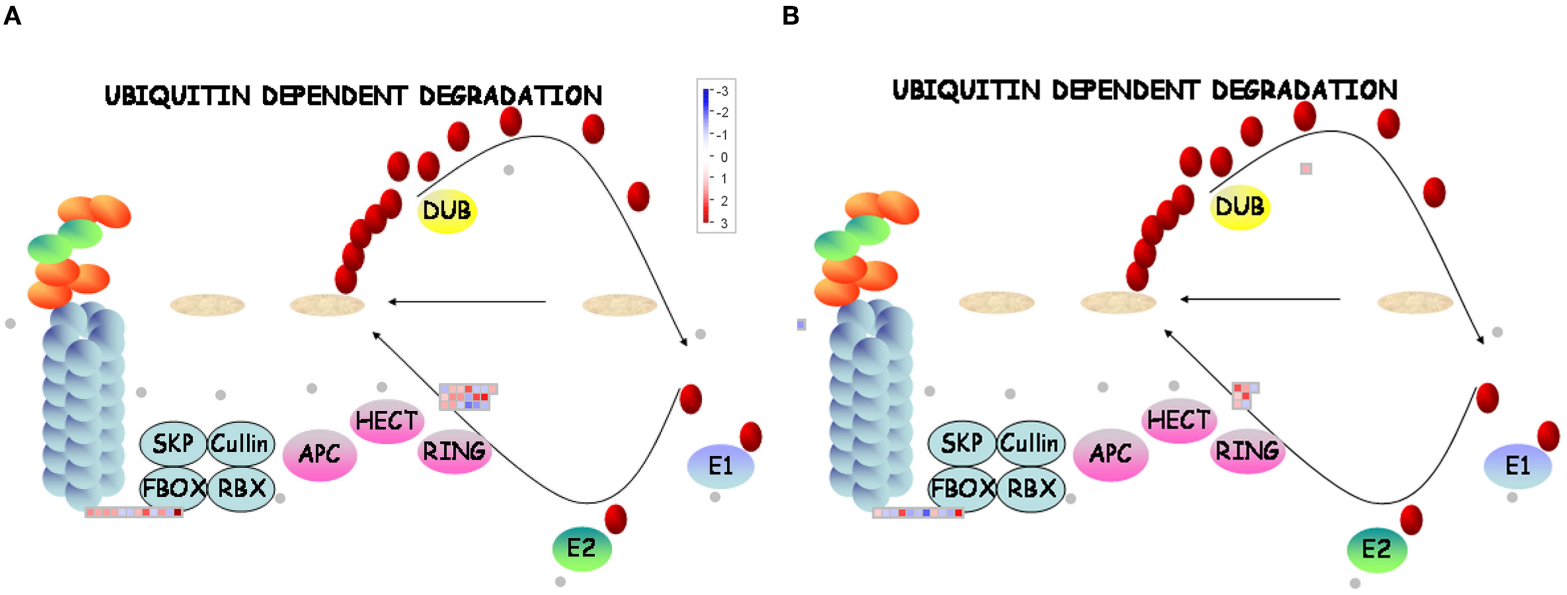
MapMan display of the coordinated changes in the expression levels of genes involved in the ubiquitin-dependent degradation pathway in different dormancy varieties of alfalfa under LT. Shown are DEGs in (D + LT)/D **(A)** and (ND + LT)/ND **(B)** under LT condition. Squares represent DEGs, red and blue indicate up- and downregulated genes, respectively. dormancy (D), non-dormancy (ND).

**Figure 7:**
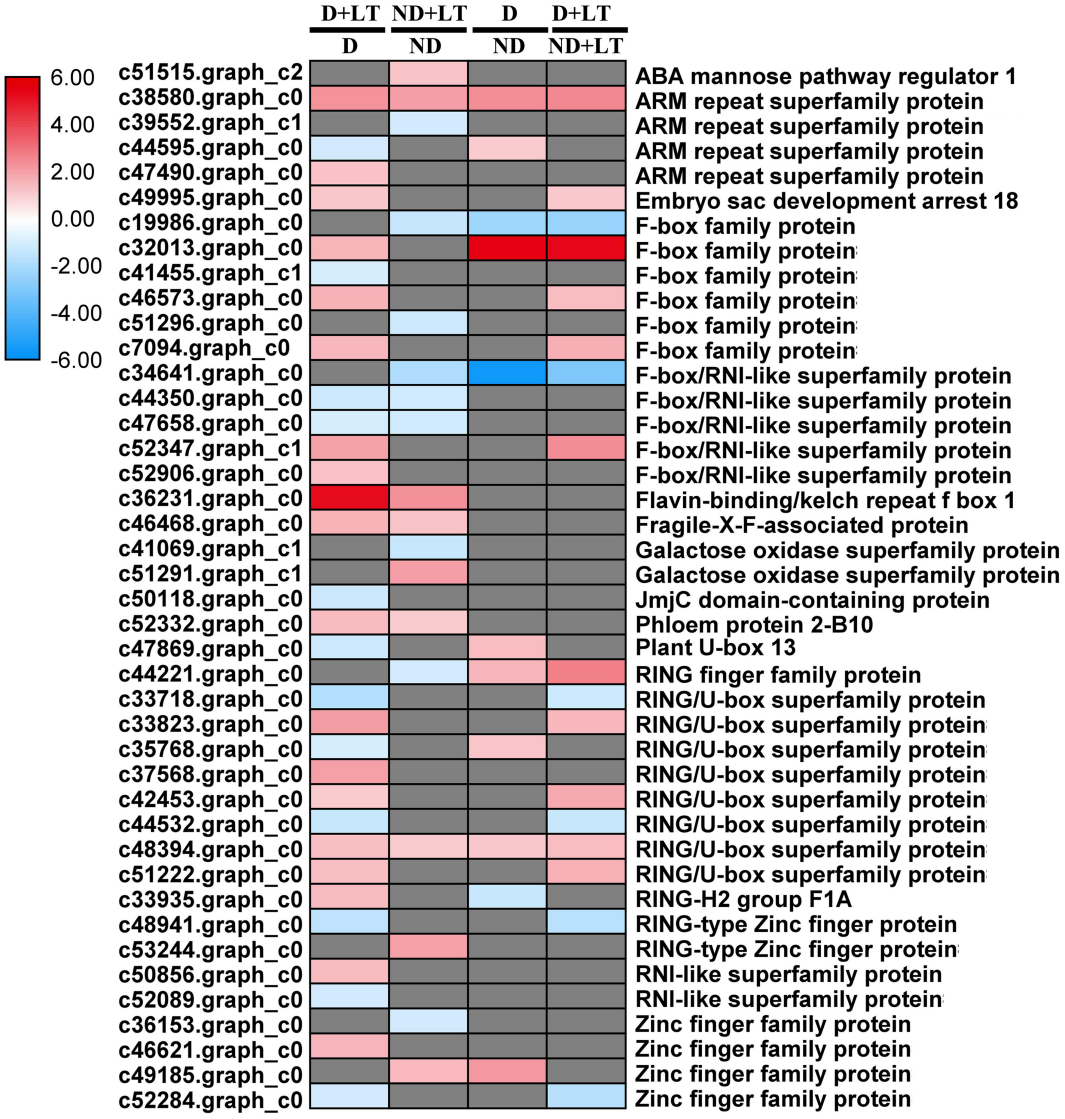
Transcript profiles of the ubiquitin-dependent degradation genes in different dormancy varieties of alfalfa under LT. The color scale indicates log_2_-transformed fold changes in expression levels between dormancy (D) and non-dormancy (ND) genotype under normal growth condition or LT condition. Red, blue, and gray denote upregulation, downregulation, and no change in expression, respectively.

A total of 31 DEGs (19 upregulated/12 downregulated) in D genotype and 18 DEGs (nine upregulated/nine downregulated) in ND genotype involved in ubiquitin-dependent degradation were significantly enriched by low temperature compared with that grown under control condition. At the same time, 11 DEGs (8 upregulated/3 downregulated) and 17 DEGs (11 upregulated/6 downregulated) involved in ubiquitin-dependent degradation were significantly enriched in D genotype compared with that in ND genotype under normal or low-temperature conditions, respectively (Figures 6A,B, 7 and Supplementary Table 3). These mainly include genes involved in *ARM repeat superfamily protein, F-box family protein, RING/U-box superfamily protein*, and *zinc finger family protein*. Together, these results revealed that more proteasome-related genes were upregulated in response to low temperature in D genotype alfalfa.

### RT-qPCR Validation of DEGs Identified by RNA-Seq

**Figure 8:**
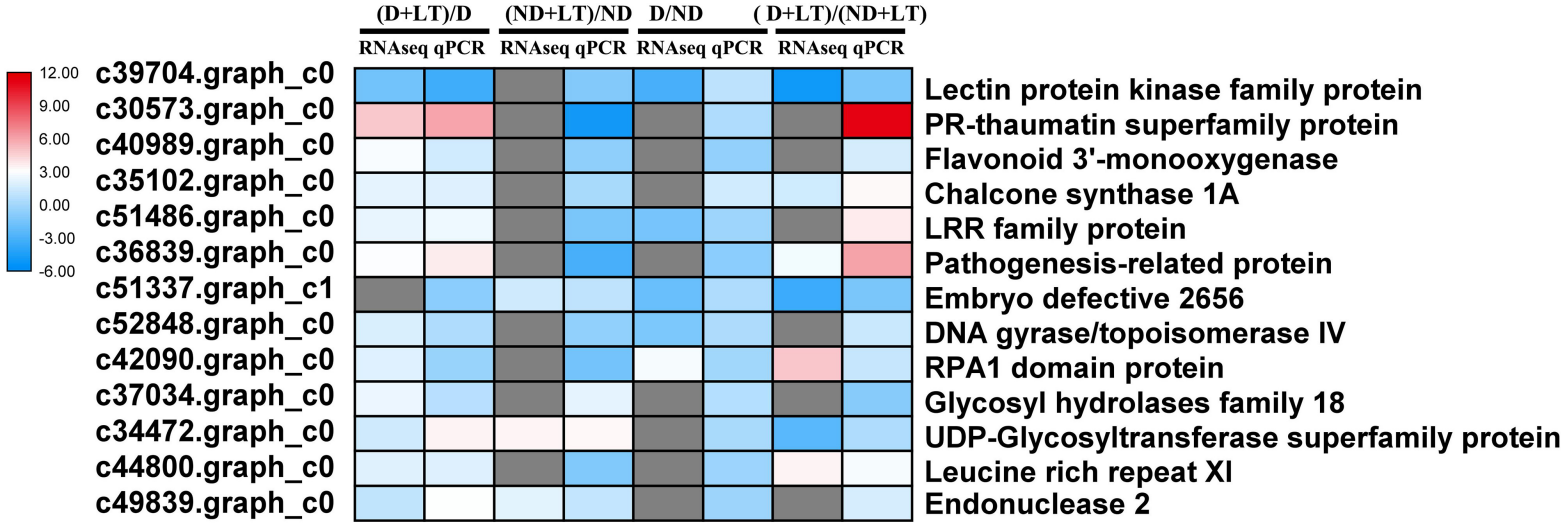
Comparison between the results of the RT-qPCR and RNA-Seq analyses of selected DEGs. The color scale indicates log_2_-transformed fold changes of gene expression levels. Red, blue, and gray denote upregulation, downregulation, and no change in expression, respectively.

To validate RNA-Seq results, 13 genes involved in flavonoids biosynthesis, receptor kinase signaling pathway, RNA and protein synthesis, and ubiquitin-dependent degradation were selected for RT-qPCR analysis. In D alfalfa samples under low temperature, 12 of the 13 DEGs had a strong correlation between qPCR and RNA-Seq data. In addition, three genes showed a strong correlation between qPCR and RNA-Seq data in ND alfalfa samples, three genes and five genes showed good agreement between D and ND under normal or low-temperature conditions, respectively (Figure 8).

## Discussion

### Dormancy of Alfalfa and Flavonoids Biosynthesis

Low temperature is a primary determinant of plant growth and survival, a positive correlation between the accumulation of flavonoids and cold tolerance was observed in Arabidopsis (Schulz et al., 2016); furthermore, the biosynthetic genes of flavonoids (*CHS, CHI, F3H, FLS*, and *ANS*) were strongly induced in cold-tolerance than cold-sensitive Arabidopsis (Hannah et al., 2006; Baskar et al., 2018). In the present study, 20 out of 37 flavonoid biosynthesis genes were identified significantly upregulated in D genotype under low-temperature treatment, among them 13 genes were exclusively upregulated in D genotype compared with ND genotype. Meanwhile, *CHS1A* (c35102.graph_c), *leucoanthocyanidin dioxygenase* (c36471.graph_c0), *flavonoid 3'-monooxygenase* (c48733.graph_c0), and *UDP-glycosyltransferase 72B1* (c50658.graph_c0, c30913.graph_c0) were also identified highly upregulated in D genotype compared with ND genotype under low temperature. A previous study also indicated that flavonoid biosynthesis genes were enriched in dormancy alfalfa during cold tolerance (Zhou et al., 2018). All these results further demonstrated upregulation of key biosynthetic genes of flavonoids played essential roles in response to low temperature in D genotype alfalfa. However, we also noticed reduced flavonoids contents in the D genotype at low temperatures. Previous studies had shown that H_2_O_2_ and ${\text{O}}_{2}^{-}$ were induced significantly when alfalfa was exposed to cold stress (Zhou et al., 2018; Cui et al., 2019), and flavonoids acted as an antioxidant to scavenge reactive oxygen species (ROS) (Baskar et al., 2018). Therefore, our study further indicated that more flavonoids were used to scavenge ROS in the D genotype, and much more detailed experiments were needed in the future to further explore the correlation between flavonoids and ROS in alfalfa.

### Dormancy of Alfalfa and Receptor-Like Kinase Family Genes

Receptor-like kinases are well-known as conserved signaling components such as extracellular ligand-binding domain, transmembrane domain, and cytoplasmic kinase domain, and play vital roles in external signal perception, activating the downstream signaling pathways and response to diverse stresses (Wrzaczek et al., 2010; Zhang et al., 2013). The CRPK1, a receptor-like cytoplasmic kinase, translocated cold signal from the plasma membrane to the nucleus and influenced freezing tolerance in Arabidopsis by fine-tuning the CBF signaling (Liu Z. et al., 2017). In the present study, 90.20% of RLKs were significantly upregulated by low temperature in genotype D compared with that grown under normal temperature. At the same time, 80.10% of RLK in genotype (D + LT)/(ND + LT) were significantly upregulated, while only 18.18% of LRK in genotype ND were upregulated under low temperature. Further analysis, *low temperature-responsive proteins* (c52776.graph_c0), *DRE transcription factors* (c51631.graph_c0), and *cold acclimation proteins* (c42469.graph_c0) in D genotype were significantly upregulated under low temperature. In agreement with these results, a previous study also demonstrated that Zebra leaf 15, a receptor-like protein kinase, was involved in moderate low-temperature signaling, and influenced the expression of downstream *OsWRKY71* and *OsMYB4* in rice (Feng et al., 2019). We thus speculate that LRKs might induce a higher expression of these downstream genes that enhance D genotype alfalfa cold tolerance.

### Dormancy of Alfalfa, RNA, and Protein Synthesis and Protein Degradation

RNA processing, protein synthesis, and protein post-translational modification play crucial roles in plant response to cold stress. In this research, approximately 75% of RNA and protein synthesis genes in the D genotype were significantly upregulated under low temperature, most of which are involved in post-translational modification (*chaperone, PP2C, MAPK/ERK kinase kinase 1*, and partially *RLKs*). However, most of these genes were not induced significantly in the ND genotype by low temperature. Interestingly, ~86% of RNA-processing genes in the ND genotype were significantly downregulated involved in RNA transcription, splicing, helicase, and ribonucleases. Consistent with our results, the rice *wsl5* (*white stripe leaf 5)* mutant that bears a mutation in an RNA binding protein, showed albinic leaves phenotype and died at low temperature (20°C) later on. Further research indicated that *wsl5* mutation impaired the editing of *rpl2* (*ribosomal protein L2*), and splicing of *rpl2* and *rps12* (*ribosomal protein S12*) (Liu et al., 2018). OST1 (OPEN STOMATA 1), a well-known Ser/Thr protein kinase, positively regulates freezing tolerance by phosphorylating the downstream cold tolerance genes *ICE1, BTF3* (basic transcription factor 3), *PUB25*, and *PUB26* in Arabidopsis (Ding et al., 2015; Chen et al., 2021). For ubiquitin-dependent protein degradation, 61.29% of ubiquitin-dependent degradation genes in D genotype were significantly upregulated by low temperature treatment, among which *the embryo sac development arrest 18* (c49995.graph_c0), *F-box family protein* (c46573.graph_c0, c7094.graph_c0), *F-box/RNI-like superfamily protein* (c52347.graph_c1), and *RING/U-box superfamily protein* (c33823.graph_c0, c42453.graph_c0 and c51222.graph_c0) were also identified highly upregulated in D genotype compared with ND genotype by low temperature treatment. The protein ubiquitination and subsequent degradation by proteasome are important processes for plant resistance to cold stress (Liu J. Y. et al., 2017). The overexpression of *OsPUB2*, a U-box E3 Ub ligase showed markedly better tolerance to cold stress associated with higher survival rates, chlorophyll content, and cold-stress-inducible genes in rice (Byun et al., 2017). Consistent with these results, 40 F-box proteins were identified as highly induced in response to freezing stress in field-grown alfalfa (Song et al., 2016). Taken together, our results indicated that the upregulated expression of genes involved in post-translational modifications and ubiquitin-dependent degradation might play pivotal roles in D genotype alfalfa cold tolerance.

## Conclusions

**Figure 9:**
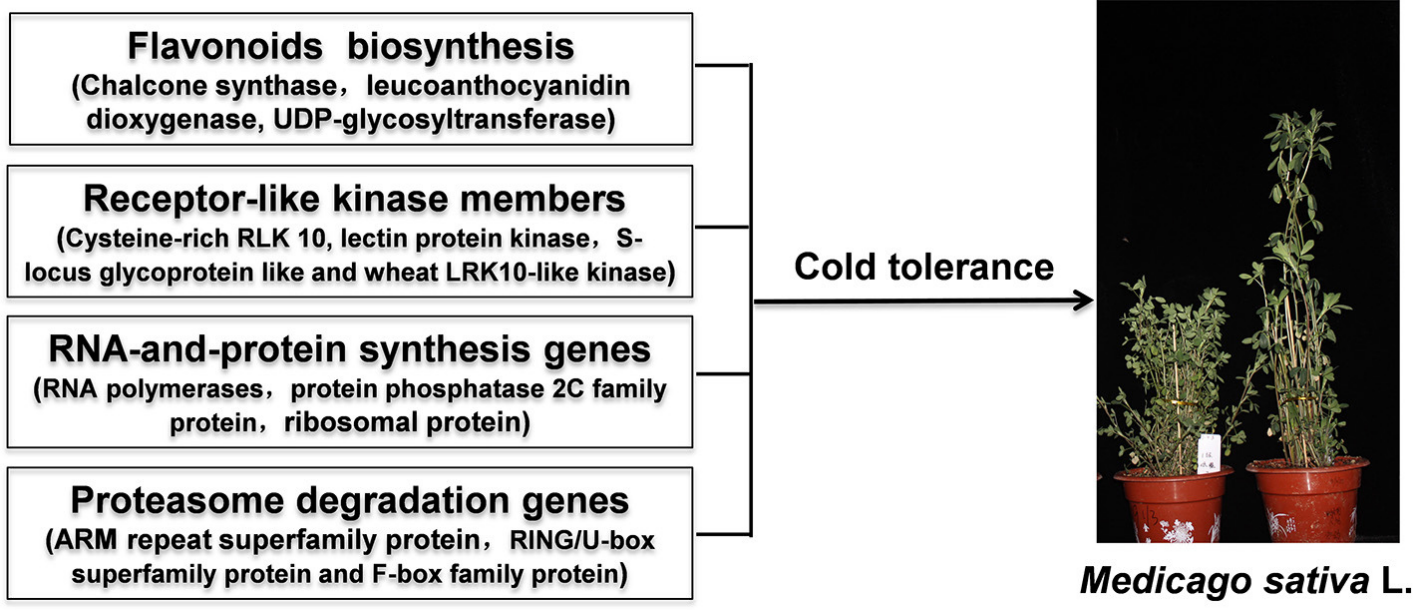
Diagram of a proposed regulatory network for low temperature tolerance in alfalfa.

Based on the phenotypical, physiological and transcriptomic analyses, flavonoids content, and higher expression of flavonoids biosynthesis genes may play essential roles in response to low-temperature stress in D genotype alfalfa. Further analyses revealed that receptor-like kinase family genes, RNA, and protein synthesis genes, and proteasome degradation pathway genes also highly upregulated expression and contributed to cold tolerance phenotype in D genotype alfalfa (Figure 9). Our results provide insights into the regulatory mechanisms underlying D genotype alfalfa cold tolerance/dormancy and offer new target genes for future functional characterization and genetic improvement of alfalfa.

## Data Availability Statement

The datasets presented in this study can be found in online repositories. The names of the repository/repositories and accession number(s) can be found at: National Center for Biotechnology Information (NCBI) BioProject database under accession number PRJNA604710.

## Author Contributions

XM, TW, and JL designed and performed the experiments. XM, TW, JL, and YWe wrote the manuscript. XM and YWa conceived the study, supervised the project, and edited the manuscript. BL, QG, WJ, and ZW assisted in performing the experiments. All authors read and approved the final manuscript.

## Funding

This research was supported by the National Natural Science Foundation of China (32171671) and the China Agriculture Research System of MOF and MARA.

## Conflict of Interest

The authors declare that the research was conducted in the absence of any commercial or financial relationships that could be construed as a potential conflict of interest.

## Publisher's Note

All claims expressed in this article are solely those of the authors and do not necessarily represent those of their affiliated organizations, or those of the publisher, the editors and the reviewers. Any product that may be evaluated in this article, or claim that may be made by its manufacturer, is not guaranteed or endorsed by the publisher.

## Supplementary Material

Supplementary Figure 1 MapMan display of the coordinated changes in the expression levels of genes involved in flavonoids biosynthesis in dormancy (D) and non-dormancy (ND) genotype alfalfa under lower temperature (LT) conditions. Shown are differentially expressed genes (DEGs) in (D + LT)/D **(A)** and (ND + LT)/ND **(B)** under LT condition. Squares represent DEGs, red and blue indicate up- and downregulated genes, respectively.

Supplementary Figure 2 MapMan display of the coordinated changes in the expression levels of genes involved in receptor-like kinase in dormancy (D) and non-dormancy (ND) genotype alfalfa under LT conditions. Shown are DEGs in (D + LT)/D **(A)** and (ND + LT)/ND **(B)** under lower temperature condition. Squares represent DEGs, red and blue indicate up- and downregulated genes, respectively.

Supplementary Figure 3 MapMan display of the coordinated changes in the expression levels of genes involved in RNA and protein synthesis in dormancy (D) and non-dormancy (ND) genotype alfalfa under LT conditions. Shown are DEGs in (D + LT)/D **(A)** and (ND + LT)/ND **(B)** under LT condition. Squares represent DEGs, red and blue indicate up- and downregulated genes, respectively.

Supplementary Table 1 qRT-PCR validation of selected DEGs identified by RNA-seq in dormancy (D) and non-dormancy (ND) genotype alfalfa under lower temperature (LT) condition.

Supplementary Table 2 Differentially expressed genes in dormancy (D) and non-dormancy (ND) genotype alfalfa under lower temperature (LT) condition.

Supplementary Table 3 Mapman pathway enrichment analysis of DEGs in dormancy (D) and non-dormancy (ND) genotype alfalfa under lower temperature (LT) condition.
