# Supplementary figures and images for: Identification and Characterization of Regulatory Pathways Controlling Dormancy Under Lower Temperature in Alfalfa (Medicago sativa L.)
Source: Front Plant Sci. 2022 Jun 2;13:872839. doi: 10.3389/fpls.2022.872839 (PMC9201922; doi:10.3389/fpls.2022.872839)

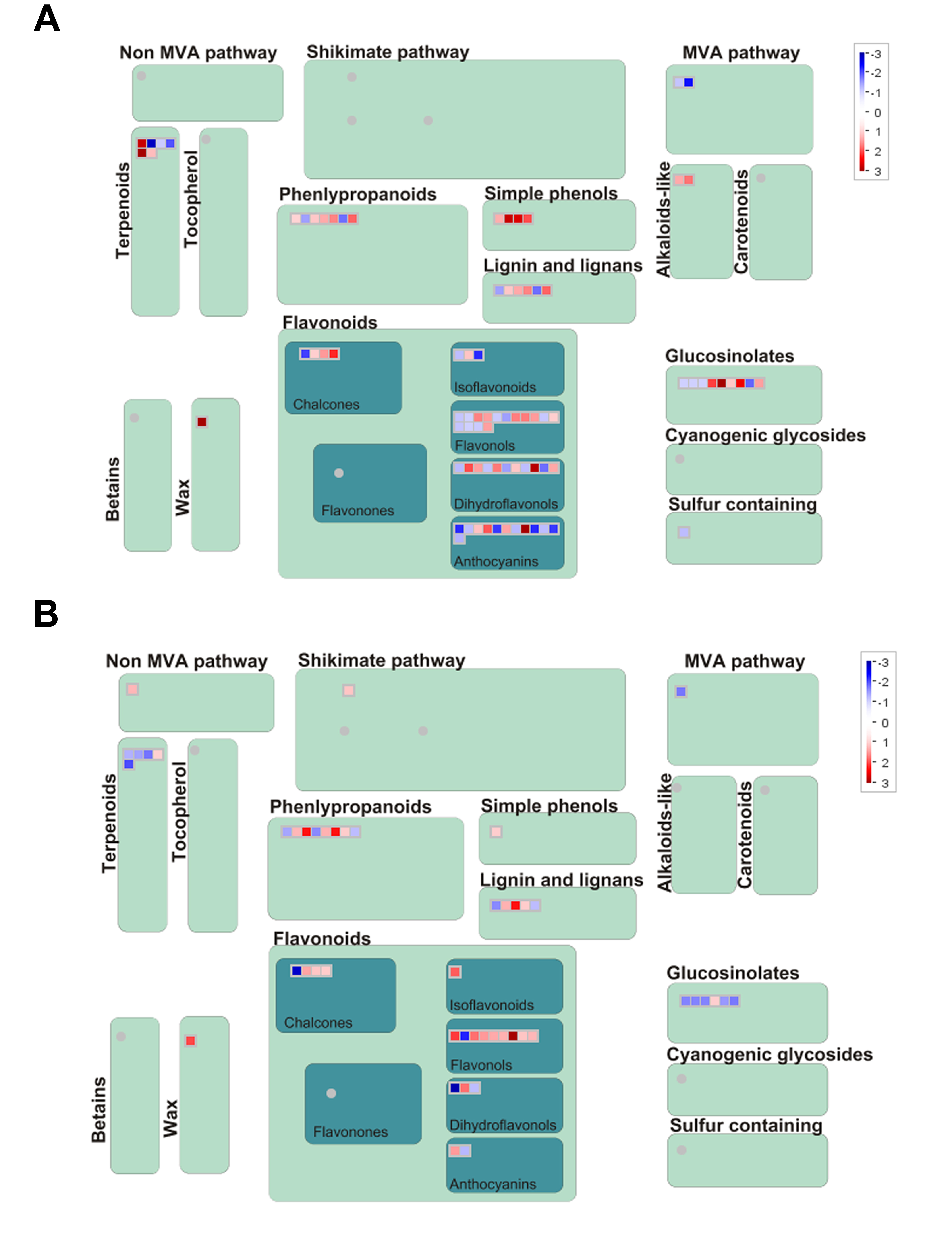

Supplement: Supplementary Figure 1 — MapMan display of the coordinated changes in the expression levels of genes involved in flavonoids biosynthesis in dormancy (D) and non-dormancy (ND) genotype alfalfa under lower temperature (LT) conditions. Shown are differentially expressed genes (DEGs) in (D + LT)/D (A) and (ND + LT)/ND (B) under LT condition. Squares represent DEGs, red and blue indicate up- and downregulated genes, respectively. [file Image_1.JPEG]

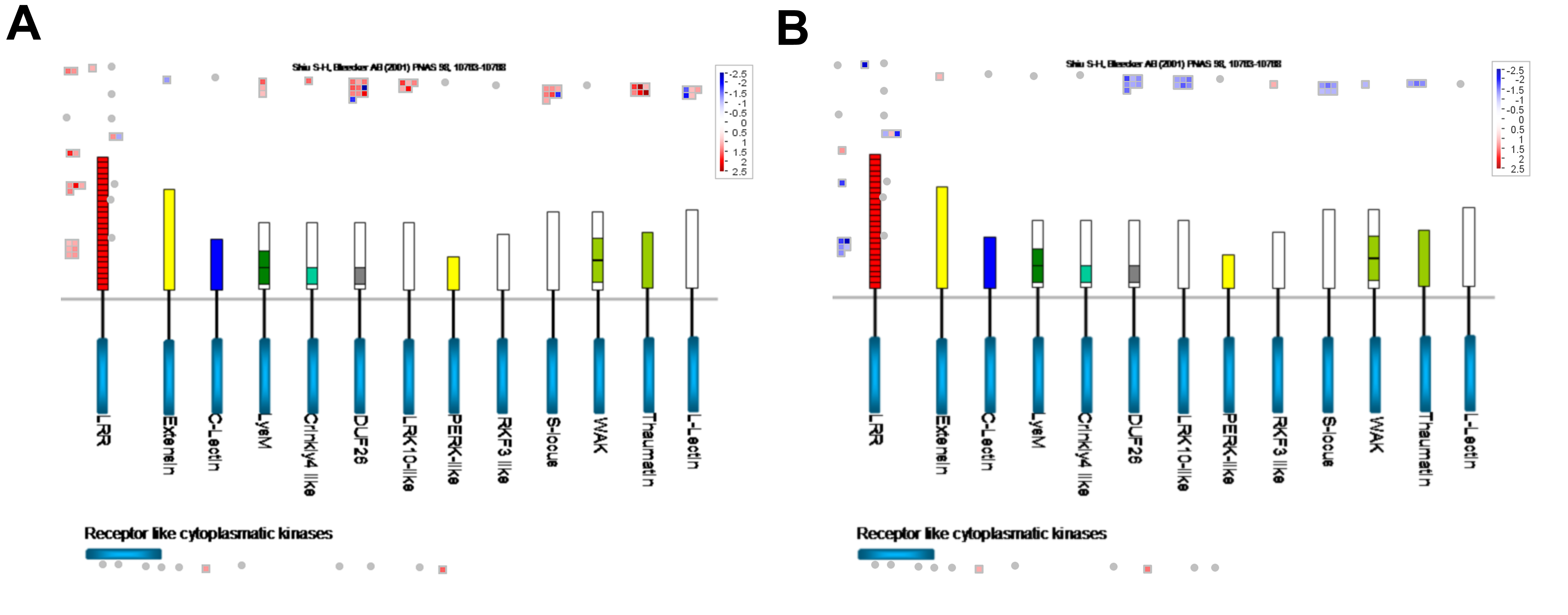

Supplement: Supplementary Figure 2 — MapMan display of the coordinated changes in the expression levels of genes involved in receptor-like kinase in dormancy (D) and non-dormancy (ND) genotype alfalfa under LT conditions. Shown are DEGs in (D + LT)/D (A) and (ND + LT)/ND (B) under lower temperature condition. Squares represent DEGs, red and blue indicate up- and downregulated genes, respectively. [file Image_2.JPEG]

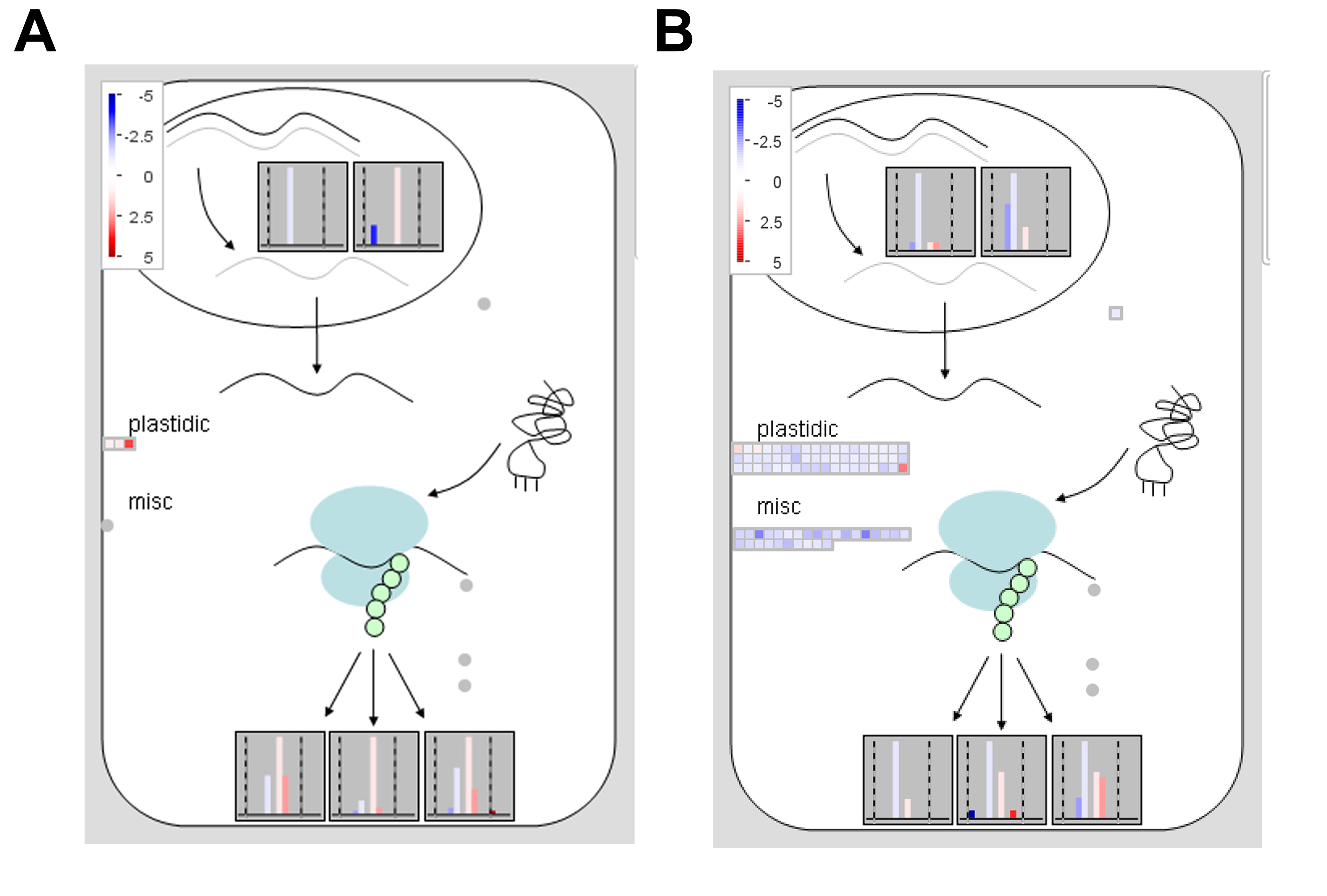

Supplement: Supplementary Figure 3 — MapMan display of the coordinated changes in the expression levels of genes involved in RNA and protein synthesis in dormancy (D) and non-dormancy (ND) genotype alfalfa under LT conditions. Shown are DEGs in (D + LT)/D (A) and (ND + LT)/ND (B) under LT condition. Squares represent DEGs, red and blue indicate up- and downregulated genes, respectively. [file Image_3.JPEG]
